# Supplementary material for: Implementation and Early Outcomes of an Antimicrobial Stewardship Program in South Korea
Source: Antibiotics (Basel). 2025 Aug 17;14(8):834. doi: 10.3390/antibiotics14080834 (PMC12382939; doi:10.3390/antibiotics14080834)
Supplement: Supplementary file 1 [file antibiotics-14-00834-s001.zip › antibiotics-3761347_supplementary table S1.pdf]

Supplementary Table S1. List of Restricted Antibiotics and ASP Intervention Criteria

Antibiotic Name	Drug Class	Common Indications	ASP Restriction Criteria
**Amphotericin B**	Polyene antifungal	Invasive fungal infections	Use restricted to confirmed/probable invasive fungal disease; ID consult recommended
**Caspofungin acetate**	Echinocandin antifungal	Candidemia, invasive candidiasis	Limited to resistant strains or intolerance to first-line agents
**Cefepime HCl**	4th-generation cephalosporin	Febrile neutropenia, HAP/VAP	Reserved for neutropenic fever or severe nosocomial infections
**Ceftazidime+Avibactam**	β-lactam/β-lactamase inhibitor	CRE infections	Use limited to confirmed carbapenemase-producing Enterobacterales
**Ceftolozane+Tazobactam**	β-lactam/β-lactamase inhibitor	MDR Pseudomonas infections	Restricted to P. aeruginosa resistant to other β-lactams
**Colistimethate sodium**	Polymyxin	XDR gram-negative infections	Requires DST confirmation of resistance to all other agents
**Daptomycin**	Lipopeptide	MRSA bacteremia, VRE infections	Reserved for proven MRSA/VRE or intolerance to vancomycin/linezolid
**Ertapenem**	Carbapenem	ESBL-producing Enterobacterales	Use restricted to ESBL-producing pathogens
**Imipenem/Cilastatin**	Carbapenem	Severe intra-abdominal or resistant infections	Use restricted to ESBL-producing pathogens
**Isavuconazole**	Triazole antifungal	Invasive aspergillosis, mucormycosis	Use permitted only in confirmed/probable fungal disease
**Linezolid**	Oxazolidinone	MRSA/VRE infections	Reserved for MRSA/VRE when vancomycin not tolerated or ineffective
**Meropenem trihydrate**	Carbapenem	Severe infections with resistant gram-negatives	Use restricted to ESBL-producing pathogens

**Micafungin sodium**	Echinocandin antifungal	Invasive candidiasis	Prior azole failure or resistance required
**Teicoplanin**	Glycopeptide	MRSA or gram-positive cocci infections	Reserved for patients with vancomycin allergy or intolerance
**Tigecycline**	Glycylcycline	MDR gram-negative and polymicrobial infections	Use limited to documented resistance or allergy to first-line therapies
**Vancomycin HCl**	Glycopeptide	MRSA, C. difficile (oral), gram-positive infections	Culture-confirmed gram-positive infection or strong clinical suspicion
**Voriconazole**	Triazole antifungal	Invasive aspergillosis	Use restricted to high-risk or confirmed fungal infections
