# Supplementary material for: Implementation and Early Outcomes of an Antimicrobial Stewardship Program in South Korea
Source: Antibiotics (Basel). 2025 Aug 17;14(8):834. doi: 10.3390/antibiotics14080834 (PMC12382939; doi:10.3390/antibiotics14080834)
Supplement: Supplementary file 1 [file antibiotics-14-00834-s001.zip › antibiotics-3761347_supplementary table S2.pdf]

Supplementary table S2. Top 5 Most Frequently Used Restricted Antibiotics (January–April 2025)

**Antibiotic**	**Jan**	**Feb**	**Mar**	**Apr**	**Total**
Meropenem trihydrate	207	162	194	222	785
Vancomycin HCl	160	85	105	171	521
Teicoplanin	92	94	87	114	387
Imipenem/Cilastatin	16	12	18	24	70
Ceftolozane+Tazobactam	9	7	18	11	45
