# Supplementary material for: Implementation and Early Outcomes of an Antimicrobial Stewardship Program in South Korea
Source: Antibiotics (Basel). 2025 Aug 17;14(8):834. doi: 10.3390/antibiotics14080834 (PMC12382939; doi:10.3390/antibiotics14080834)
Supplement: Supplementary file 1 [file antibiotics-14-00834-s001.zip › antibiotics-3761347_supplementary table S3.pdf]

Supplementary Table S3: Monthly DOT by Restricted Antibiotics (Jan–Apr 2025)

Antibiotic	Jan	Feb	Mar	Apr
Amphotericin B	12	1	4	6
Caspofungin acetate	0	0	0	0
Cefepime HCl	0	0	0	0
Ceftazidime+Avibactam	20	6	7	14
Ceftolozane+Tazobactam	3	7	18	11
Colistimethate sodium	51	27	24	25
Daptomycin	0	0	0	1
Ertapenem	11	16	6	6
Imipenem/Cilastatin	14	12	18	24
Isavuconazole	0	0	3	0
Linezolid	5	5	15	9
Meropenem trihydrate	227	162	194	222
Micafungin sodium	19	8	15	19
Teicoplanin	83	94	87	114
Tigecycline	2	13	11	5
Vancomycin HCl	152	85	105	171
Voriconazole	26	13	14	13
