# Supplementary figures and images for: Implementation and Early Outcomes of an Antimicrobial Stewardship Program in South Korea
Source: Antibiotics (Basel). 2025 Aug 17;14(8):834. doi: 10.3390/antibiotics14080834 (PMC12382939; doi:10.3390/antibiotics14080834)

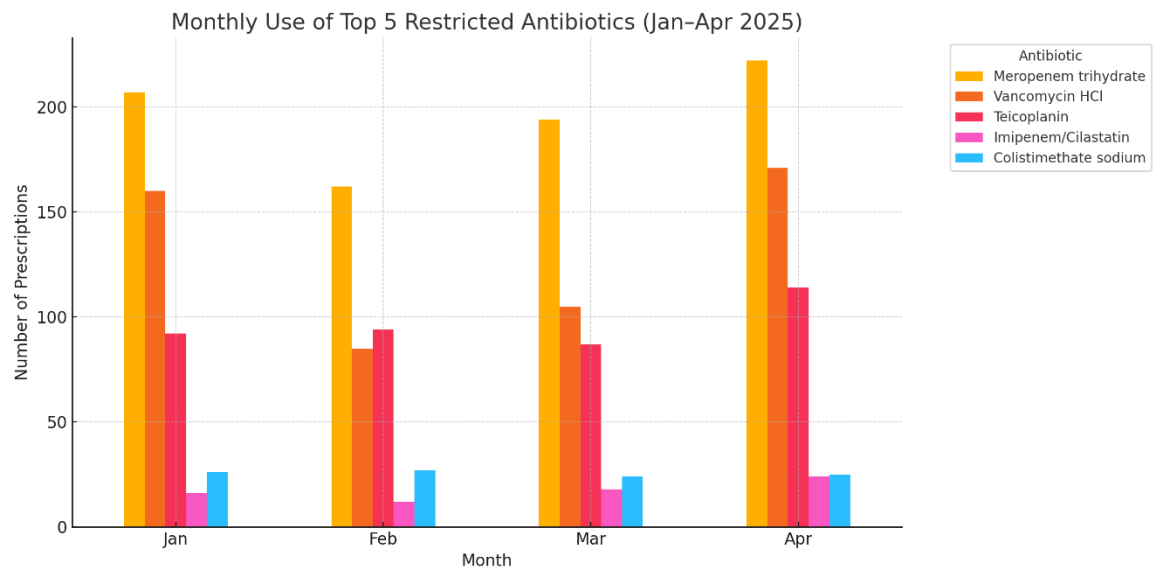

Supplement: Supplementary file 1 [file antibiotics-14-00834-s001.zip › antibiotics-3761347_supplementary figure S1.pdf]
